# Supplementary material for: How dentists and oral and maxillofacial surgeons deal with tooth extraction without a valid clinical indication
Source: PLoS One. 2023 Jan 17;18(1):e0280288. doi: 10.1371/journal.pone.0280288 (PMC9844881; doi:10.1371/journal.pone.0280288)
Supplement: S1 File — (DOCX) [file pone.0280288.s001.docx]

*Questionnaire*

Non-dental reasons for tooth removal

For most questions, you are expected to provide only one answer. However, more answers are possible to some questions. If this is the case, it will be stated with the relevant question. Read through all the answer options before ticking the number next to the answer of your choice. If you made a mistake, cross out the wrong answer and give the intended answer. Finally, for some questions, no answer options are given, but you are requested to write down an answer or explanation yourself.

| 1 | Since January 2016, have you ever received a request from patients for the extraction of one or more teeth on non-dental (for example psychological, cultural, financial, or other) grounds? | | |
| --- | --- | --- | --- |
|  | 1 | Yes | |
|  | 2 | No | *Go to question 14* |

| 2 | How many requests on non-dental grounds do you estimate to have received in the period from January 2016 to now? | |
| --- | --- | --- |
|  | . . . . | *(please enter number)* |

| 3 | *Listed below are several reasons based on which patients make an extraction request on non-dental grounds.*  For each reason, can you indicate by how many of the patients – who made an extraction request to you in the period from January 2016 to now – that was mentioned?  *[more reasons possible per patient]*  ***Example****: Suppose three patients made an extraction request, the first one because of phobic fear, the second one because of financial reasons, and the third one because of financial and also cultural reasons. Your response would then be: ‘1’ at A, ‘2’at D, and ‘1’ at E.* | | |
| --- | --- | --- | --- |
|  | A | Severe dental fear | . . . . patient(s) |
|  | B | Unexplained pain | . . . . patient(s) |
|  | C | Morphodysforic disorder^1^ | . . . . patient(s) |
|  | D | Financial reasons | . . . . patient(s) |
|  | E | Cultural reasons | . . . . patient(s) |
|  | F | Other reason, namely: | . . . . patient(s) |
| 1. *Morphodysforic disorder: a body dysmorphic disorder in which certain parts of the body (e.g., ‘teeth’) are found to be horrible, while the environment cannot discover anything special about it* | | | |

*Below are more questions* ***about the most recent extraction request*** *you received on non-dental grounds.*

| 4 | What reason(s) did the patient have for the extraction request?  *(More than one answer possible)* | |
| --- | --- | --- |
|  | 1 | Severe dental fear |
|  | 2 | Unexplained pain |
|  | 3 | Morphodysforic disorder |
|  | 4 | Financial reasons |
|  | 5 | Cultural reasons |
|  | 6 | Other reasons, namely: |

| 5 | In deciding whether or not to grant the extraction request, was it assessed whether the patient was competent to make decisions about his/her dental care? | | |
| --- | --- | --- | --- |
|  | 1 | Yes | |
|  | 2 | No | *Go to question 7* |

| 6 | If so, in what way(s) did this happen at the time?  *(More than one answer possible)* | |
| --- | --- | --- |
|  | 1 | By asking (open) test questions to the patient, such as: ‘What could be the consequences if you have your tooth/teeth removed?’ |
|  | 2 | In consultation with the family/spouse |
|  | 3 | In consultation with a doctor |
|  | 4 | In consultation with a psychologist/psychiatrist |
|  | 5 | In consultation with a colleague (dentist/oral surgeon) |
|  | 6 | In another way, namely: |

| 7 | If not, why didn’t this happen?  *(More than one answer possible)* | |
| --- | --- | --- |
|  | 1 | I don’t know how to do that |
|  | 2 | The time pressure does not allow that |
|  | 3 | If the patient was not mentally competent, someone would have accompanied him |
|  | 4 | The reference didn’t say anything about incompetency |
|  | 5 | The patient does not live in an institution, so I have assumed that he is mentally competent |
|  | 6 | Other reasons, namely: |

| 8 | Have you fulfilled the extraction request? | | |
| --- | --- | --- | --- |
|  | 1 | Yes | |
|  | 2 | No | *Go to question 14* |

| 9 | Have you had any doubts about that decision? | | |
| --- | --- | --- | --- |
|  | 1 | Yes | |
|  | 2 | No | *Go to question 11* |

| 10 | Which aspects of the extraction request did you have doubts about at the time?  *(More than one answer possible)* | |
| --- | --- | --- |
|  | 1 | If it was allowed to perform these extractions |
|  | 2 | If the patient would later regret the extraction |
|  | 3 | If there were no alternatives |
|  | 4 | Another aspect, namely: |

| 11 | Did you regret your decision to comply with the extraction request afterward? | |
| --- | --- | --- |
|  | 1 | Yes |
|  | 2 | No |

| 12 | Did the patient regret the extraction(s) afterward? | | |
| --- | --- | --- | --- |
|  | 1 | Yes | |
|  | 2 | No | *Go to question 14* |
|  | 3 | I don’t know. There was no contact with the patient anymore | *Go to question 14* |

| 13 | For what reason did the patient regret his decision?  *(More than one answer possible)* | |
| --- | --- | --- |
|  | 1 | I don’t know, because the patient didn’t tell |
|  | 2 | The patient now experiences dental problems, that he didn’t expect |
|  | 3 | The patient knows that there are less drastic solutions for his problem |
|  | 4 | The patient’s environment appeals to him about his decision |
|  | 5 | The patient experiences disadvantages/discomfort from the lack of natural teeth |
|  | 6 | Other reason, namely: |

| 14 | Below is a brief description of a case of a patient requesting an extraction on non-dental grounds. Would you grant this patient’s request or not?  *Johan has had a huge dental fear for years. He only visits the dentist when he is in pain. He is 35 years old. His teeth are pretty good, according to the dentist. Yet he has been wanting to have his teeth pulled out for years so that he never has to undergo scary dental treatments again. An anxiety treatment has already been offered to him, but he does not feel that he will get rid of his anxiety by this.* | | |
| --- | --- | --- | --- |
|  | 1 | Yes, I would grant the request | |
|  | 2 | No, I would not grant the request | Go to question 16 |
|  | 3 | I don’t know if I would grant the request | Go to question 17 |

| 15 | Based on which consideration(s) would you grant the request?  *(More than one answer possible)* | |
| --- | --- | --- |
|  | 1 | I am convinced that he made a well-considered decision |
|  | 2 | I think the patient should be able to decide for themselves |
|  | 3 | In my opinion, there is no other way to help him properly |
|  | 4 | If I don’t perform the extraction, another dentist or oral surgeon will do it |
|  | 5 | Other consideration, namely: |

| 16 | Based on which consideration(s) would you not grant the request?  *(More than one answer possible)* | |
| --- | --- | --- |
|  | 1 | There is a good alternative using a treatment aimed to reduce fear of dental treatment |
|  | 2 | It is an irreversible treatment |
|  | 3 | I am ethically bound to not cause unnecessary damage to a patient’s teeth |
|  | 4 | The teeth can still be saved |
|  | 5 | The patient is insufficiently aware of the situation because of his dental fear |
|  | 6 | Another reason, namely |

| 17 | *Below are some statements regarding extraction requests from patients on non-dental grounds.*  Please tick the extent to which you agree or disagree with each of them. | | | | | |
| --- | --- | --- | --- | --- | --- | --- |
|  | *fully disagree* | *mostly disagree* | *not disagree, not agree* | *mostly agree* | *fully agree* | *no opinion/ not applicable* |
| a | Over the years, I have become less reluctant to grant an extraction request on non-dental grounds. | | | | | |
|  | 1 | 2 | 3 | 4 | 5 | 6 |
| b | With extraction on a non-dental basis, I do not provide a patient with good care. | | | | | |
|  | 1 | 2 | 3 | 4 | 5 | 6 |
| c | In my experience, patients with non-dental requests for extraction usually do no regret the extraction afterward. | | | | | |
|  | 1 | 2 | 3 | 4 | 5 | 6 |
| d | I have learned better which alternatives I can offer to patients who request an extraction on non-dental grounds | | | | | |
|  | 1 | 2 | 3 | 4 | 5 | 6 |
| e | I think it is a bad thing that one colleague does accept an extraction request from a patient on non-dental grounds and the other colleague does not. | | | | | |
|  | 1 | 2 | 3 | 4 | 5 | 6 |
| f | If it concerns an extraction request from a patient on non-dental grounds, I consider myself bound by the idea that the patient may make his own decision in the context of personal autonomy. | | | | | |
|  | 1 | 2 | 3 | 4 | 5 | 6 |
| g | There should be clearer guidelines on whether or not to grant patient requests for extraction on non-dental grounds. | | | | | |
|  | 1 | 2 | 3 | 4 | 5 | 6 |
| h | A patient’s request for extraction on a non-dental basis is, by definition, a complex care issue. | | | | | |
|  | 1 | 2 | 3 | 4 | 5 | 6 |
